# A Rare Case of De Novo Acute Myeloid Leukemia, Featuring a *KMT2A* (*MLL*) Amplification

**DOI:** 10.3390/diagnostics16060820

**Published:** 2026-03-10

**Authors:** Fares Hassan, Jeff Chen, Charles Westphal, Carlos A. Tirado

**Affiliations:** 1 The International Circle of Genetics Studies Project, New York Chapter, 25 Pinnacle Dr., New York, NY 11777, USA; fares.hassan@stonybrook.edu (F.H.); jeff.chen.2@stonybrook.edu (J.C.); charles.westphal@stonybrook.edu (C.W.); 2 Department of Biology, Stony Brook University, Stony Brook, NY 11794, USA; 3 Department of Pathology, Stony Brook University Hospital, Stony Brook, NY 11794, USA; 4 The Cytogenetics Lab, Department of Pathology, Stony Brook University Hospital, Stony Brook, NY 11794, USA

**Keywords:** acute myeloid leukemia, myeloid, MLL, complex karyotype, MYC, FISH, secondary-related AML, adverse-risk

## Abstract

We present a case of a patient in their 80s initially presenting with myelodysplastic syndromes (MDS). Chromosomal analysis showed an abnormal female karyotype with a complex karyotype. Metaphase FISH confirmed four copies of *KMT2A (MLL)* in 24.5% [49/200] and amplification of *KMT2A* (*MLL*) with more than four copies in 22% [44/200]. FISH also revealed the presence of *MYC* (8q24) on the long arm of chromosome 2 at 2q33 locus, two copies of *BCR* on each homolog 22, and two additional copies of *BCR* on a derivative chromosome 22. Flow cytometric analysis revealed a population of aberrant myeloid blasts (15–17%). Bone marrow analysis showed hypercellular marrow with a significant increase in myeloid blasts (~50%) and trilineage dysplasia. Eventually, these findings were consistent with a final diagnosis of acute myeloid leukemia non-M3 and a complex karyotype, correlating with cytogenetics, flow cytometry, molecular, and clinical findings. The patient’s clinical course was marked by a rapid deterioration, including recurrent arrhythmias, hypoxic respiratory failure, and septic shock. Given their poor clinical status and adverse-risk molecular profile, care was transported to hospice. The presence of *KMT2A* amplification is a rare event in AML and is present in ~1% of AML and MDS cases. *MYC* translocation, *KMT2A* (*MLL*) amplification, and 5q/20q losses suggest secondary therapy-related AML and categorize this case in the adverse risk prognosis under the ELN 2022 guidelines.

## 1. Introduction

Acute myeloid leukemia (AML) is a heterogeneous hematologic malignancy characterized by uncontrolled clonal proliferation of immature myeloid precursors [1]. Meanwhile, myelodysplastic syndromes (MDS) are similar, involving ineffective hematopoiesis in bone marrow neoplasms [2]. The difference lies in a blast-count boundary; if the patient has less than 20% blasts, they are considered MDS, and if they have more than 20% blasts, they are considered AML [3]. However, this is not a hard absolute, and depending on the patient’s case, they can be classified as MDS or AML, regardless of being above or under a 20% blast count.

AML is characterized by clonal expansion of immature myeloid precursor cells in the bone marrow and peripheral blood, disrupting normal hematopoiesis [4]. It affects primarily patients >65 years with an overall survival (OS) of 32% (<10% in patients >60 years) [5,6,7]. Furthermore, AML comprises 15–20% of pediatric leukemia and 35% of adult leukemia along with numerous chromosomal abnormalities commonly reported, contributing to its poor prognosis [8]. MDS is sometimes referred to as preleukemia, given its ability to transform into leukemias such as AML. This is known as secondary AML, or AML-MR, which is much more complex than AML [9].

Although AML is most common de novo, development through AML-MR, prior cytotoxic therapy, or environmental exposures is also well documented. AML-MR typically involves complex cytogenetic abnormalities and is associated with an adverse prognosis under the ELN 2022 guidelines [10,11]. *MYC* is a well-studied oncogenic driver of cellular proliferation, differentiation inhibition, and other roles in myeloid malignancies. Rearrangements and amplifications of *MYC* are well studied in lymphoid malignancies but MYC translocations are exceedingly rare in AML and, when found, are associated with therapy-related myeloid neoplasms or relapses [12,13]. The most common *KMT2A* rearrangement is the reciprocal translocation, partnering with >130 genes and chimeric fusion proteins seen in acute lymphoid leukemia (ALL), MDS, and AML [14]. *KMT2A* is a common mutation in AML, found in 3–10% of adult AML, and found to be an aggressive and prognostically unfavorable leukemic marker. *KMT2A*-r disrupts epigenetic regulation through the formation of chimeric proteins that activate oncogenic signaling pathways such as HOXA and MEIS1 [14].

However, *KMT2A* amplification, in contrast to rearrangement, is exceedingly rare, occurring in approximately 1% of AML and MDS cases and carrying a particularly poor prognosis associated with low response to chemotherapy and extremely short survival [15]. The presence of *KMT2A* amplification in AML is seen more frequently in elderly patients and coexists with other high-risk abnormalities, including highly complex karyotypes, del5q, *TP53* loss or mutation, and occasionally del7q. Furthermore, data have suggested that *KMT2A* amplification has a pathogenesis different from *KMT2A*-r, further complicating its role as a rare genetic abnormality [16]. Amplification of *KMT2A* in AML was first observed in 1999 as although extra copies of *KMT2A* were previously recorded in the literature, true gene amplification was not verified [17]. Then, in a 2015 study involving 21 patients with *KMT2A* amplification in AML/MDS (11 with AML-MR, six therapy-related AML, and four therapy-related MDS), again highly complex karyotypes, *TP53* deletion and mutation, and del5q were frequently observed. Furthermore, all patients demonstrated resistance to therapy with a median OS of 1 month after *KMT2A* amplification detection [18].

In this report, we present a case of AML with a *KMT2A* amplification, *MYC* translocation, and a complex karyotype. We will provide a summary of the case presentation, diagnostic workup, outcome, and insight into the management of the patient. We will conclude with a thorough discussion of the abnormalities discovered in this report and similar cases involving *KMT2A* amplifications and *MYC* rearrangements. Although *KMT2A* amplifications, found in 1% of AML and MDS, and *MYC* translocations are two notably rare events in their own sense, they are exceedingly rare in co-occurrence with very few reports in the literature, highlighting the importance of this case [16].

## 2. Case Presentation

A woman in her 80s with a history of coronary artery disease status post ST-elevation myocardial infarction and coronary artery bypass grafting, hypertension, hyperlipidemia, and a complicated postoperative sternal wound infection presented for evaluation of tachycardia. She was noted to have atrial fibrillation with rapid ventricular response and dyspnea on exertion following prolonged travel. Workup for pulmonary embolism was negative, and she was admitted for cardiac monitoring. Review of prior laboratory data showed normal blood counts before cardiac surgery.

Her postoperative course following coronary artery bypass grafting was complicated by recurrent wound infections and bacteremia requiring multiple surgical debridements and prolonged antibiotic therapy over the ensuing weeks. During this postoperative period, new-onset cytopenias were first noted. On admission, laboratory evaluation demonstrated pancytopenia with a white blood cell count of 2.56 × 10^9^/L (ANC 1.26 × 10^9^/L), hemoglobin 6.8 g/dL (requiring transfusion of one unit of packed red blood cells), and platelet count of 57 × 10^9^/L. Hematology was consulted for the evaluation of pancytopenia. Initial workup revealed no evidence of hemolysis, nutritional deficiency, or monoclonal gammopathy (vitamin B12 in the 400s pg/mL, folate 14 ng/mL, and iron studies were consistent with inflammation, with an elevated ferritin of 420 ng/mL). The cytopenias were initially attributed to chronic inflammation and recent infection.

Given that the patient demonstrated clinical stability and slow improvement in blood counts, management options, including observation versus bone marrow biopsy, were discussed, with a shared decision to pursue close monitoring and supportive care. However, after approximately two months of persistent pancytopenia without hematologic recovery, a bone marrow aspiration and core biopsy were performed.

A complete blood count (CBC) obtained in July of 2025 demonstrated a white blood cell count (WBC) of 3.00 k/µL, hemoglobin of 9.2 g/dL, hematocrit of 29.5%, mean corpuscular volume of 91.6 fL, and a platelet count of 83 k/µL. Evaluation of bone marrow was performed, including aspiration, core biopsy, touch preparation, and clot section. The aspirate and touch-prep are markedly hypercellular and demonstrate trilineage arrest with an increase in blasts. Histological analysis demonstrated that blasts are medium to large in size with moderate cytoplasm and absent cytoplasmic granules and no Auer rods. Dysplastic changes were present in all three hematopoietic lineages, with the myeloid lineage showing neutrophils with hypogranular forms as well as abnormally lobate forms. The erythroid lineage displayed megablastoid maturation, and megakaryocytes showed hypolobated nuclei. A 200-cell bone marrow aspirate differential showed markedly increased blasts and increased nucleated red blood cells along with reduced mature myeloid forms (Table 1).

Flow cytometric analysis revealed a population of aberrant myeloid blasts (about 15–17% of total events). The cells express CD45 (dim+), CD34 (+), CD117 (+), CD33 (dim+), CD13 (+), CD123 (weak+), HLA-DR (+), CD38 (variable negative to dim positive), CD7 (small subset, variable dim+), CD4 (−), CD11b (−), CD15 (−), CD11c (weak/−), CD135 (+), and MPO (+). The marrow was markedly hypercellular (80–90%) with clusters of myeloblasts and mild marrow fibrosis (grade 0–1). Iron staining revealed increased ring sideroblasts.

The patient was initially planned for hypomethylating agent–based therapy but experienced rapid clinical deterioration, including recurrent atrial arrhythmias, hypoxic respiratory failure, neutropenic fever, pneumonia, and septic shock requiring intensive care. Care was transitioned to a comfort-focused approach, and the patient was discharged to hospice.

## 3. Materials and Methods

### 3.1. Conventional Cytogenetics

Chromosome analysis was performed using conventional cytogenetics protocols, including trypsin-Giemsa (G-band) banding of twenty metaphase spreads, eleven from a 24 h bone marrow culture and nine from a 72 h culture at a resolution of 400 chromosome bands. Results were annotated according to the International System for Human Cytogenomic Nomenclature 2024 standards [19]

### 3.2. Molecular Cytogenetics

Fluorescence in situ hybridization (FISH) was performed using the following probes: XL *KMT2A* BA (11q23.3) break-apart, XL *MECOM* (3q26), XL t(15;17) Dual Fusion (15q24, 17q21.1-q21.2) (MetaSystems, Medford, MA, USA), LSI 5q *EGR1*/D5S23, D5S721 (5q31, 5p15.2), LSI D7S486/CEP7 (7q31/7p11.1-q11.1 [D7Z1]), LSI *RUNX1*/*RUNX1T1* (8q21.3, 21q22), LSI *MYC* DC BAR (8q24), LSI *BCR*/*ABL1* DC DF (9q34, 22q11.2) (Abbott, Des Plaines, IL, USA), LSI *CBFB* (16q22) DC BAR, LSI *CEP8* (D8Z2), LSI *BCL2* (18q21) BAR, *PDGFRA* (4q12) BA (Empire Genomics, Depew, NY, USA), and del(20q) Deletion (20q12, 20q13.1) (CytoCell, Cambridge, UK).

This case report was conducted in accordance with institutional ethical standards and the Declaration of Helsinki. Due to the patient’s death during the same hospitalization and the absence of identifiable next of kin, written informed consent specific to publication could not be obtained. However, a general consent for treatment and use of de-identified clinical information for educational and research purposes had been signed at the time of hospital admission. All identifying information has been removed to protect patient privacy.

## 4. Results

### 4.1. Flow Cytometry and Blast Assessment

The immunophenotypic profile suggests a clonal population of immature myeloid blasts showing abnormal maturation. Altogether, the biopsy findings provide important information not fully realized within the initial flow cytometry and aspirate differential. Flow cytometry identified 15–17% aberrant myeloid blasts, and the aspirate differential showed a diagnostically significant blast percentage, while the performed core biopsy and CD34/CD117 immunostains demonstrated a markedly higher blast burden of approximately 40–50%. The blast burden of approximately 40–50% on core biopsy was estimated based on morphologic assessment of representative sections along with CD34 and CD117 immunohistochemical staining. Given the discrepancy between modalities, the highest blast percentage identified on core biopsy was used for the final disease classification, as the aspirate and flow cytometric assessments were felt to underestimate blast burden due to sampling limitations.

### 4.2. Conventional Cytogenetics

**Figure 1:**
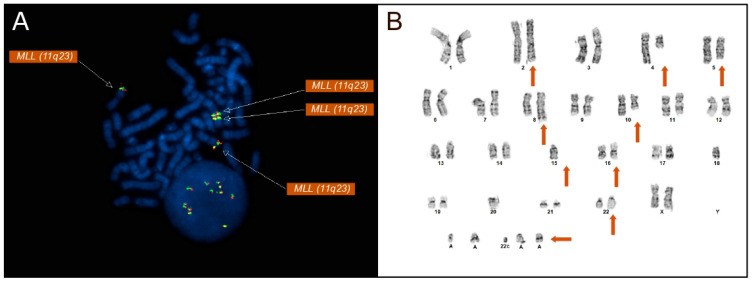
(**A**) Metaphase FISH using a KMT2A (MLL, 11q23) break-apart probe demonstrating multiple KMT2A signals, including additional amplified KMT2A material on chromosome 16p13. The presence of multiple KMT2A signals indicates gene copy number amplification rather than a balanced chromosomal rearrangement. All KMT2A signals on the metaphase FISH are denoted by arrows. (**B**) The patient’s G-banded karyotype displays a complex karyotype including multiple structural and numeric abnormalities (orange arrows denote derivative chromosomes displaying abnormalities). A complex karyotype is defined by the presence of multiple structural and numerical chromosomal abnormalities. Additional material involving chromosomes 2, 5, 8, 10, 16, and 22, as well as marker chromosomes, is indicated.

Conventional cytogenetics utilizing the 20 Giemsa-banded metaphase spreads demonstrated an abnormal female karyotype with a highly complex pattern of structural and numerical abnormalities (Figure 1B). The composite karyotype was described as 45~48,XX,add(2)(q33),add(4)(q13),add(5)(q22),add(8)(q24),add(10)(q22),add(16) (p13.3), 18,−20,add(22)(q13),+add(22)(q13),+1~5 mar[cp20]ish. The findings were consistent with a complex karyotype characterized by multiple unbalanced rearrangements.

### 4.3. Molecular Cytogenetics

FISH analysis was performed on 200 nuclei utilizing a panel of probes targeting recurrent AML and MDS-associated abnormalities. Most notably, FISH analysis with the *KMT2A* (*MLL*) break-apart probe revealed an increased copy number of *KMT2A* signals. Four copies of *KMT2A* were found in 24.5% [49/200] of nuclei, and more than four copies were found in 22% [44/200], displaying an amplification of *KMT2A* not commonly observed in AML and MDS cases (Figure 1A). FISH analysis confirmed the presence of an amplification of *KMT2A* rather than the more commonly associated rearrangements seen in AML and MDS patients.

Additionally, the FISH results were consistent with one copy of *EGR1* in 83.5% [167/200] of nuclei. Similarly to *KMT2A*, four copies of *BCR* were found in 88% [176/200] of the examined nuclei. Additionally, one copy of *BCL2*, *PTPRT*, and *MYBL2* was found in 85.5% [171/200], 81% [162/200], and 81% [162/200], respectively.

**Figure 2:**
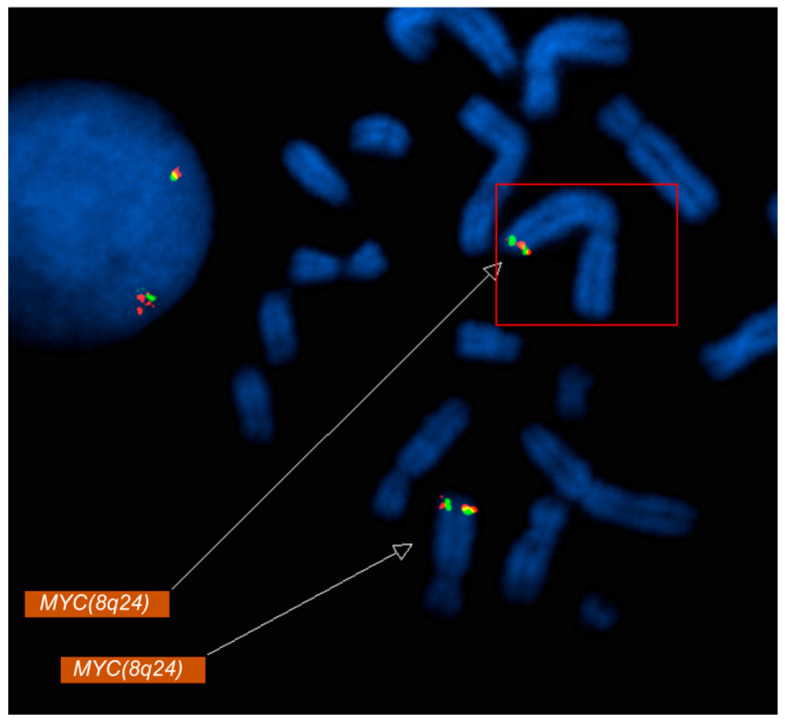
Metaphase FISH analysis using a MYC (8q24) probe demonstrates relocation of MYC signals to the long arm of chromosome 2 (2q33). Fluorescent MYC signals (red/green) are visualized on metaphase chromosomes (DAPI counterstain in blue). The boxed region and upper arrow highlight the aberrant MYC signal on chromosome 2q33, consistent with a MYC rearrangement/translocation, while the lower arrow indicates the MYC signal at the native 8q24 locus.

Metaphase FISH showed the relocation of *MYC* signals to the long arm of chromosome 2 at 2q33 (Figure 2), along with loss of 5q31 on one homolog of chromosome 5 (Figure 1B). Metaphase FISH also revealed two additional *KMT2A* signals localized to chromosome 16p13, indicating insertion of amplified *KMT2A* material at this locus (Figure 1A). Similarly, two copies of *BCR* were identified on each normal chromosome 22, with two additional *BCR* signals present on a derivative chromosome 22, consistent with the *BCR* copy number gain. Additionally molecular testing identified a *TP53* mutation.

Deletion probes targeting 5q and 20q displayed a loss of material consistent with a report of del(5q) and del(20q). No evidence of t(15;17)(q24;q21), *RUNX1*::*RUNX1T1*, *CBFB* rearrangement, *MECOM* rearrangement, *PDGFRA* rearrangement, or *BCL2* rearrangement was identified.

## 5. Discussion

*KMT2A* is located on chromosome 11q23 and catalyzes the methylation of lysine residues on histone tails, particularly histone H3 typically at lysine 4. Unlike other epigenetic enzymes like histone acetyltransferases (HATs), KMTs modify just one or two lysines per histone [20,21]. It is primarily the SET domain at the C-terminus of KMTs that is responsible for methylation of H3 at lysine 4 (H3K4). In previous studies, *KMT2A* has been shown to be part of a macro protein complex that functions to promote stability of *KMT2A* and regulate transcriptional activation of *HOX* genes [21,22]. *KMT2A* amplifications, in contrast to translocations, are defined as focal copy number amplifications that result in increases in gene copy number of the *KMT2A* gene [23]. Furthermore, mutations involving balanced rearrangements of *KMT2A* (*KMT2A*-r) have been commonly observed in AML, appearing in 3–10% of AML patients, while *KMT2A* amplifications are much rarer in AML [14,16].

*MYC* is a proto-oncogene located at chromosome 8q24, playing roles in cell-cycle progression, apoptosis, and cellular transformation [24,25]. As an oncogene, *MYC* is one of the most frequently mutated events in cancer, including one of the most frequently amplified regions in AML [25]. In recent studies, *MYC* has shown to regulate downstream genes responsible for apoptosis and differentiation of AML cells as well as being overexpressed/required for myeloid leukemias triggered by *FLT3*-ITD and PML-RARA and other fusion oncoproteins [26]. In de novo AML patients, overexpression of *MYC* has been associated with inferior survival outcomes with more limited clinical data on *MYC* rearrangements in AML patients though some studies have revealed chemotherapy resistance [25,26].

This patient’s case highlights many unique features of a complex karyotype present with atypical abnormalities such as *KMT2A* amplifications and translocations of *MYC* in AML. As a patient in her 80s, this falls under the observation that *KMT2A* amplifications, if seen, are typically present in elderly patients with AML or MDS [16]. This patient’s prior history of major surgery and coronary heart disease, recurring infections, and subsequent prolonged antibiotic therapy all contribute to the comorbidities that may have affected the eventual prognosis of this patient.

Bone marrow evaluations helped reveal a significantly hypercellular marrow with trilineage dysplasia and a large increase in myeloid blasts, demonstrating progression to AML rather than MDS and establishing a final diagnosis of acute myeloid leukemia (non-M3). The presence of ring sideroblasts and mild marrow fibrosis further illustrates the degree of marrow dysregulation in this patient.

The differences in blast percentages reported across flow cytometry (15–17%), bone marrow aspirate differential (26%) and core biopsy (40–50%), illustrate an important clinical discordance. This is a common discrepancy in AML, reflecting sampling variability and differences in methodology, and is important to recognize. These differences are critical as relying on a single assessment may underestimate disease significance or delay diagnosis and risk stratification, even misclassifying a patient (AML v. MDS). Integrating all morphological and immunophenotypic data is a necessity in ensuring an accurate evaluation of blast burden and guiding appropriate decision making.

Cytogenetic and molecular findings in this patient also demonstrate the aggressive nature of her disease. *KMT2A* amplification co-occurred with a *MYC* translocation to chromosome 2q33, *TP53* was found to be mutated, and there were marked losses of 5q and 20q. While *BCR* copy number gains were also observed, they are deemed to likely be less clinically relevant than the *MYC* and *KMT2A* abnormalities. *MYC* translocations combined with amplification of *KMT2A* in an AML patient are an exceptionally rare occurrence. Coupled with the complex karyotype of the patient and the *TP53* mutation found present, these indicators effectively foreshadowed an extremely poor prognosis, especially when the age and history of the patient are considered. These findings correlate closely with the patient’s rapid clinical deterioration, including recurrent atrial arrhythmias, hypoxic respiratory failure, neutropenic fever, and ultimately the transition to hospice care.

In 2022, karyotyping/FISH data on 42 AML/MDS patients and next-generation sequencing (NGS) and prognostic data on a patient subset was studied, all with *KMT2A* amplification [27]. With a median age at diagnosis of 70 years, the median survival was 45 days and a complex karyotype was found in 100% (*n* = 38) of cases. 5q and 17p deletions were the most common findings followed by del7q and gain of chromosome 8 with *TP53* mutations, the most commonly found in NGS [27]. As seen in previous studies, AML/MDS with *KMT2A* amplification was associated with poor prognosis and specific cytogenetic abnormalities including complex karyotypes, del5q, and *TP53* mutations. *MYC* translocations are also seen to carry a significantly adverse prognosis, as in one study involving t(3;8)(q26.2;q24) patients (8 with t-AML, 3 with t-MDS), a common *MYC* translocation, 18 out of 20 patients died with a median survival of ~6 months [28].

Gray et al. in 2023 then demonstrated that histone H3 lysine 9 mono- and di-methylation (H3K9me1/2) balance at the *KMT2A* locus can regulate these amplifications and rearrangements [29]. This balance, through crosstalk between the depletion of lysine demethylase KDM3B that increases H3K9me1/2 levels and methyltransferase G9a/EHMT2, increases amplification of *KMT2A*. It was seen that depleting CFTC, a transcription factor, at the *KMT2A* locus through KDM3B depletion was also enough to generate these genetic aberrations [29].

This case demonstrated the challenges of managing a high-risk elderly and frail patient diagnosed with AML. Despite initial stability and careful monitoring, the patient’s disease progressed rapidly, limiting the feasibility of hypomethylating agent therapy. Along with the patient’s presentation of a complex karyotype, elderly age, and features suggestive of secondary or therapy-related AML, the selection of FISH targets was driven by their relevance in AML and MDS. *KMT2A* amplification, though rare, is generally associated with patients with similar molecular profiles. Evaluating *KMT2A* among other prognostic markers such as a *TP53* mutation provides a more comprehensive risk stratification. Furthermore, compared to previously reported cases of *KMT2A* amplified AML, our case is distinguished by the concurrent presence of a *MYC* translocation. Although *KMT2A* amplification carries an adverse outcome, typically along a complex karyotype and *TP53* mutations, a *MYC* translocation is exceptionally rare. This co-occurrence is rarely seen in the literature, underscoring the unique molecular profile of this patient.

The correlation seen between the patients’ cytogenetic and molecular abnormalities, their aggressive clinical course, and poor prognosis demonstrates the importance of early diagnostic evaluations and risk stratification in other similar patients. Integration of flow cytometry, bone marrow morphology, conventional cytogenetics, and FISH/molecular testing provided a detailed understanding of the patient’s high-risk disease biology, which helps to inform clinical decision-making and counseling in future cases with similarly rare abnormalities.

Although the current prognosis of AML with *KMT2A* aberrations is poor, there are emerging therapeutic strategies that may hold promise. For example, menin inhibitors that disrupt the interaction between menin and *KMT2A*, and suppress aberrant HOX/MEIS1 expression, have shown promising preclinical applications [14]. Revumenib and Ziftomenib, among other menin inhibitors, are being evaluated in phase I/II studies and demonstrate reduction in leukemic blasts in patient cohorts.

## 6. Conclusions

In conclusion, *KMT2A* amplifications occur in just 1% of AML/MDS cases and are functionally distinct from *KMT2A* rearrangements. Furthermore, *MYC* rearrangements are similarly rare in AML and also confer a poor prognosis. Although there are limited cases in the literature of these reports, *KMT2A* amplifications in AML have consistently been observed alongside complex karyotypes, multiple chromosomal abnormalities, and poor survival outcomes. Along with the characteristic low median OS and high resistance to therapy, the unique co-occurrence of a *MYC* rearrangement with *KMT2A* amplification is a rare case that illustrates the importance of *KMT2A* amplifications as a prognostic and therapeutic marker for AML.

## 7. Limitations

This report is limited by its single-case design; thus the findings may not be generalizable to a larger population of *KMT2A*-amplified AML patients. Given the rarity of this molecular profile, it can be difficult to directly compare results to similar patients. Longitudinal molecular monitoring was not performed, limiting assessment of clonal evolution over time. Additionally, the patient’s rapid clinical decline prevented further evaluation of treatment response or resistance patterns. Finally, given the highly complex karyotype, it is also difficult to determine the individual contribution of each cytogenetic marker to the overall course and prognosis.

## Figures and Tables

**Table 1:** Bone marrow aspirate differential (200-cell count).

Cell Type	Patient %	Reference Range %
Blasts	26.0	0–3
Promyelocytes	6.0	3–5
Myelocytes	7.0	8–17
Metamyelocytes	8.0	10–25
Band/PMN neutrophils	2.0	16–27
Eosinophils	2.0	1–6
Lymphocytes	1.0	10–23
Plasma cells	1.0	0–2
Monocytes	1.0	0–3
Nucleated red blood cells	42.0	14–30

## Data Availability

The original contributions presented in this study are included in the article. Further inquiries can be directed to the corresponding author.
